# Identification and Validation of NK Marker Genes in Ovarian Cancer by scRNA-seq Combined with WGCNA Algorithm

**DOI:** 10.1155/2023/6845701

**Published:** 2023-04-25

**Authors:** Xin He, Weiwei Feng

**Affiliations:** Department of Obstetrics and Gynecology, Ruijin Hospital, Shanghai Jiao Tong University School of Medicine, 197 Ruijin 2nd Road, Huangpu District, Shanghai 200025, China

## Abstract

**Background:**

As an innate immune system effector, natural killer cells (NK cells) play a significant role in tumor immunotherapy response and clinical outcomes.

**Methods:**

In our investigation, we collected ovarian cancer samples from TCGA and GEO cohorts, and a total of 1793 samples were included. In addition, four high-grade serous ovarian cancer scRNA-seq data were included for screening NK cell marker genes. Weighted gene coexpression network analysis (WGCNA) identified core modules and central genes associated with NK cells. The “TIMER,” “CIBERSORT,” “MCPcounter,” “xCell,” and “EPIC” algorithms were performed to predict the infiltration characteristics of different immune cell types in each sample. The LASSO-COX algorithm was employed to build risk models to predict prognosis. Finally, drug sensitivity screening was performed.

**Results:**

We first scored the NK cell infiltration of each sample and found that the level of NK cell infiltration affected the clinical outcome of ovarian cancer patients. Therefore, we analyzed four high-grade serous ovarian cancer scRNA-seq data, screening NK cell marker genes at the single-cell level. The WGCNA algorithm screens NK cell marker genes based on bulk RNA transcriptome patterns. Finally, a total of 42 NK cell marker genes were included in our investigation. Among which, 14 NK cell marker genes were then used to develop a 14-gene prognostic model for the meta-GPL570 cohort, dividing patients into high-risk and low-risk subgroups. The predictive performance of this model has been well-verified in different external cohorts. Tumor immune microenvironment analysis showed that the high-risk score of the prognostic model was positively correlated with M2 macrophages, cancer-associated fibroblast, hematopoietic stem cell, stromal score, and negatively correlated with NK cell, cytotoxicity score, B cell, and T cell CD4+Th1. In addition, we found that bleomycin, cisplatin, docetaxel, doxorubicin, gemcitabine, and etoposide were more effective in the high-risk group, while paclitaxel had a better therapeutic effect on patients in the low-risk group.

**Conclusion:**

By utilizing NK cell marker genes in our investigation, we developed a new feature that is capable of predicting patients' clinical outcomes and treatment strategies.

## 1. Introduction

In terms of incidence, ovarian cancer (OV) ranks second only to cervical cancer and uterine cancer among female reproductive system tumors [1]. OV has a very high recurrence rate and mortality, which seriously threatens women's health. Due to the lack of effective screening tools and early diagnosis difficulties, 80% of OV patients are diagnosed at an advanced stage, 50-70% of patients will relapse within 2 years after treatment, and a 5-year poor survival rate of 30% [2]. Despite recent improvements in treatment, improvements in 5-year survival rates were minimal. A new therapeutic target is needed to improve the clinical outcomes of OV patients in light of the limitations of OV treatment [3]. For this reason, the development of predictive models and the identification of new biomarkers are crucial for predicting clinical outcomes and the effects of therapeutic interventions.

In response to tumor growth, a complex microenvironment surrounds tumor cells, including stromal cells, extracellular matrix molecules, and cytokines [4]. Accumulated evidence suggested that tumor microenvironment (TME) components were thought to play a vital role in tumorigenesis and progression. Moreover, abnormal changes in TME can serve as biomarkers for immunotherapy in addition to affecting patients' prognoses [5]. Antitumor immunity has focused mainly on adaptive T cell responses, without adequate attention being given to innate immune cells. Cancer cells are rapidly recognized and killed by innate immune cells, known as NK cells [6]. As NK cells interact with target cells, their antitumor effect depends entirely on the balance between their inhibitory and activating receptors [7]. In the early stages of tumor growth, NK cells can suppress tumor invasiveness by directly destroying tumor cells and promoting adaptive T cell responses to contribute to antitumor immunity [8]. Tumor progression is controlled by both NK and T cells, which indicates that these immunocytes play a vital role in shaping antitumor immunity. NK cells in peripheral blood are reduced, which increases the risk of malignant tumors [9, 10]. Furthermore, higher numbers of tumor microenvironment NK cells component are significantly associated with better outcomes. In view of the important role of NK cells in immune antitumor, cumulative studies have explored the molecular characteristics of NK cells in cancer [11], but little was known about the comprehensive molecular mechanism of NK cells in OV patients. With the advent of single-cell RNA sequencing (scRNA-seq) technology and related analytical methods, the possibility of identifying the molecular profiles of different immune cell subsets in TME has become a reality [12]. Previous investigations have demonstrated that investigating transcriptome characteristics based on the molecular profile of immunocytes extracted from scRNA-seq information may be an effective weapon for predicting clinical outcomes and immunotherapy response [13]. Here, we investigated the comprehensive molecular mechanisms of NK cells from OV patients based on scRNA-seq data.

WGCNA is a technique for examining the variations in gene expression among several samples. The association between modules and clinical profiles can also be analyzed by clustering genes according to similar transcriptome profiles in modules (such as the immune score of patients) [14]. According to the WGCNA algorithm, this study assumed that the gene expression network obeyed the scale-free distribution and constructed the gene coexpression network. Therefore, we calculated dissimilarity coefficients between nodes in order to construct a hierarchical clustering tree. The modules were further visualized by assigning corresponding genes to different modules based on gene similarity. In our study, we investigated the expression profiles of signature genes of NK cells based on single-cell sequencing data to identify their biomarker genes and to identify core module genes associated with NK cells by WGCNA public expression analysis. Next, prediction models were developed based on these factors to predict the clinical outcome of OV by combining bulk RNA-seq datasets. In addition, the performance of the prediction model was validated with four independent cohorts, and the relationship between the prediction model and the response to chemotherapy in OV patients was investigated. These results will help us to better understand the molecular mechanisms of ovarian cancer progression.

## 2. Materials and Methods

### 2.1. Data Collection

An analysis of The Cancer Genome Atlas (TCGA) database was conducted in order to obtain RNA sequencing (RNA-seq) fragments per kilobase million (FPKM) and complete follow-up information on 372 samples. Somatic mutation data came from TCGA database. Using the “tmb” algorithm in the maftools package, each sample's tumor mutation burden (TMB) value was calculated. We performed log2 [(TPM)+1] conversion on the above raw data. In addition, we included three GPL platforms (GPL570: GSE19829, GSE18520, GSE9891, GSE26193, GSE30161, and GSE63885; GPL96: GSE3149, GSE23554, GSE26712, and GSE14764; and GPL7759: GSE13876). A total of 11 GEO cohorts and 1793 samples were included in our investigation.

Single-cell transcriptional profiling data and clinical information from ovarian cancer patients were obtained from the GEO website under accession number GSE184880, and scRNA-seq data from a total of four high-grade serous ovarian cancer samples were included.

### 2.2. Identification of NK Cell Marker Genes Based on the scRNA-seq Database

For single-cell data, we filtered cells with unique feature counts >5000 or <200 and cells with mitochondrial counts >5%. Then, the feature-expression measurements for each cell were normalized by the total expression using the default parameters of the Seurat “NormalizeData” function. Finally, all cell data were fed into a combined Seurat object via the Harmony package. Then, variable genes were scaled and the principal component (PC) was analyzed. Via “RunUMAP” function min. (Dist = 0.2 and neighbors = 20) and the “FindClusters” function (resolution = 0.5) using significant pc (top 15) for UMAP analysis and clustering. For identifying cell types, we employed automated annotation; SingleR is an automated annotation method for single-cell RNA sequencing (scRNA-seq) data [15]. Given a sample reference dataset (single cell or batch size) with known labels, it marks new units in the test dataset based on similarity to the reference. Thus, for reference datasets, the burden of manually interpreting clusters and defining marker genes only needs to be done once, while this biological knowledge can spread to new datasets in an automated manner. Differentially expressed genes (DEGs) were calculated for each cell subgroup using Wilcoxon-Mann–Whitney test in FindAllMarkers function. NK cells were calculated using three methods: EPIC algorithm, xCell algorithm, and MCPcounter algorithm, which were performed based on the “IOBR” package [16]. Adjusted *p* values < 0.01 and |log2 (fold change)| > 1 were identified as NK cell marker genes.

### 2.3. WGCNA Network Construction and Module Identification

Subsequently, we made R “WGCNA” package for coexpression network analysis of NK cell marker genes. WGCNA can be used to find clusters (modules) of highly correlated genes, summarize such clusters using module signature genes or hub genes within nodules, associate modules with each other and external sample traits (using signature gene network methods), and calculate module membership metrics [14]. Associated networks facilitate network-based gene screening methods that can be used to identify candidate biomarkers or therapeutic targets [14]. Our first step was to cluster the samples in order to determine if there were any outliers. Secondly, the coexpression network was constructed by using the automatic network construction function. The soft threshold power was calculated with the R function “pickSoftThreshold” and the coexpression similarity for adjacency calculations was increased. Third, clustering and dynamic tree-cutting functions were used to detect modules using hierarchical clustering. As a fourth step, the significance of genes and the membership of modules were calculated in order to correlate them with immune features. To further analyze the module gene information, the corresponding module gene information was extracted. Finally, we visualize the feature gene network.

### 2.4. Construction and Verification of Prognostic Model Based on NK Cell Marker Genes

Subsequently, we developed a prognostic model based on the NK cell standard genes selected by WGCNA. To minimize overfitting, prognostic genes were evaluated by LASSO Cox proportional hazards regression using the “glmnet” package [17]. LASSO is a popular high-dimensional predictive regression method widely used for survival analysis of Cox proportional hazards regression models [18]. In order to select the best model, 10-fold cross-validation was performed using the function “cv.” Finally, we used multivariate Cox regression analysis to calculate the prognostic value of specific genetic characteristics based on genes provided by LASSO Cox regression analysis. Risk models were constructed based on gene mRNA expression and risk coefficients. Risk scores were calculated using the following formula:

(1) $$\begin{array}{c} \text{riskScore}={\text{Coef}}_{1}\times \text{gene expressio}{\text{n}}_{1}+{\text{Coef}}_{2}\times \text{gene expressio}{\text{n}}_{2}+\cdots {\text{Coef}}_{n}\times \text{gene expressio}{\text{n}}_{n.} \end{array}$$

Coef represents the prognostic value of each gene in multivariate Cox regression analysis. Gene expression values represent the expression values of the corresponding model genes. Patients were divided into low-risk and high-risk groups according to the median cut-off of their risk score. R “survival” software package is a tool for statistical analysis and visualization of survival data and is widely used in scientific research work [19]. The performance of the prognostic model was validated using survival analysis on four datasets using the R package “survminer.”

### 2.5. Pathway and Functional Enrichment Analysis

According to the whole genome annotation package (org.Hs.eg.db), GO and KEGG enrichment analyses were employed to explore the obtained NK cell marker genes. Through the latest online KEGG database, “ClusterProfiler” function obtained pathway data and performs functional analysis [20]. *p* < 0.05 was considered significant.

### 2.6. Enrichment Analysis of Immune Cell Infiltration

The “TIMER,” “CIBERSORT,” “MCPcounter,” “xCell,” and “EPIC” algorithms are all favorable tools for machine learning and are used to assess cell abundance and cell-type-specific gene expression patterns from a large number of tissue transcriptome profiles, quantify the tumor immune background through the type and density of tumor-infiltrating immune cells, and are widely used in scientific research work [21–24]. In addition, the “ESTIMATE” algorithm was used to calculate the proportion of stromal components and immune components in each sample microenvironment. The levels of immunomodulators in each risk group were presented by box plot.

### 2.7. Statistical Analysis

In order to compare categorical variables between different risk groups, Wilcoxon *t*-test was used. The significance threshold was set at 0.05. For data analysis and graphic generation, R tool (version 3.6.2) was conducted.

## 3. Results

**Figure 1:**
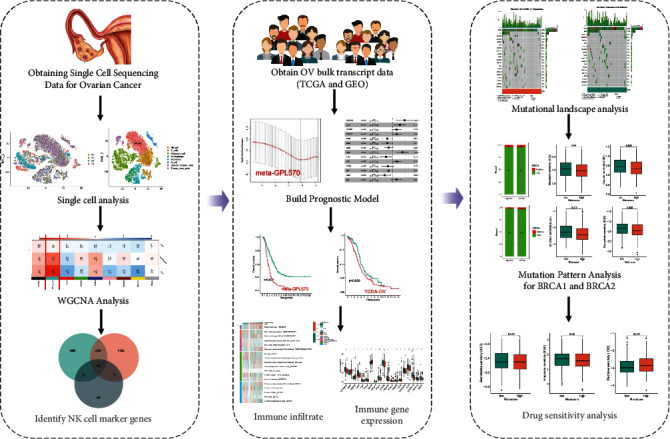
Flowchart.

The flowchart for this article is shown in Figure 1.

### 3.1. Screening of NK Cell Marker Genes Profile

**Figure 2:**
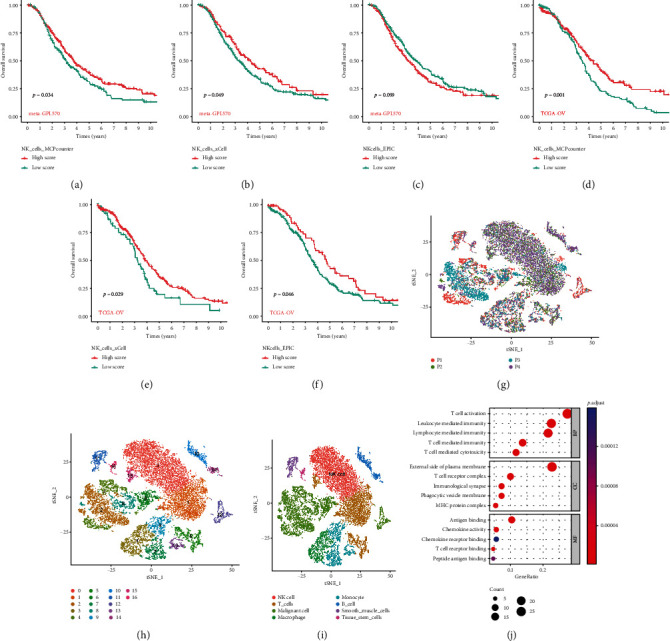
The single-cell RNA sequencing analysis identifies NK cell marker genes. (a–f) K-M survival curves suggest a prognostic role for NK cell-related scores assessed based on MCPcount, xCell, and EPIC algorithms. (g) The T-SNE algorithm demonstrated the distribution of cell subsets in four high-grade serous ovarian cancers. (h) The cell types identified by marker genes. (i) T-SNE plot colored by various cell clusters. (j) Histogram of GO analysis based on NK cell marker genes.

First, we used MCPcounter, xCell, and EPIC algorithms to calculate the NK cell index (NK score) of each sample. Based on the median score, patients were classified into high-score and low-score groups. In the meta-GPL570 cohort, NK cell infiltration contributed to the longer survival times of the high-score group than the low-score group (Figures 2(a)–2(c)). In the TCGA-OV queue, the trend is consistent with the meta-GPL570 queue (Figures 2(d)–2(f)). Based on the GSE184880 scRNA-seq data, we included four high-grade serous ovarian cancer scRNA-seq data for further investigation (Figure 2(g)). We used the first 1,500 variable genes for PCA to reduce dimensionality and then identified 17 cell clusters (Figure 2(i)). Annotating each cluster using the human primary cell map reference data set, cluster 0 was identified as NK cells using the reference data set (Figure 2(h)). There was also a difference in gene expression profiles within the cluster, and the differentially expressed genes (DEGs) for each cell subset were calculated using the Wilcoxon-Mann–Whitney test in the FindAllMarkers function. Functional enrichment showed that NK cell marker genes were mainly related to T cell immune characteristics, such as T cell activation, T cell-mediated immunity, and T cell receptor binding (Figure 2(j)).

### 3.2. Construction of Gene Coexpression Module

**Figure 3:**
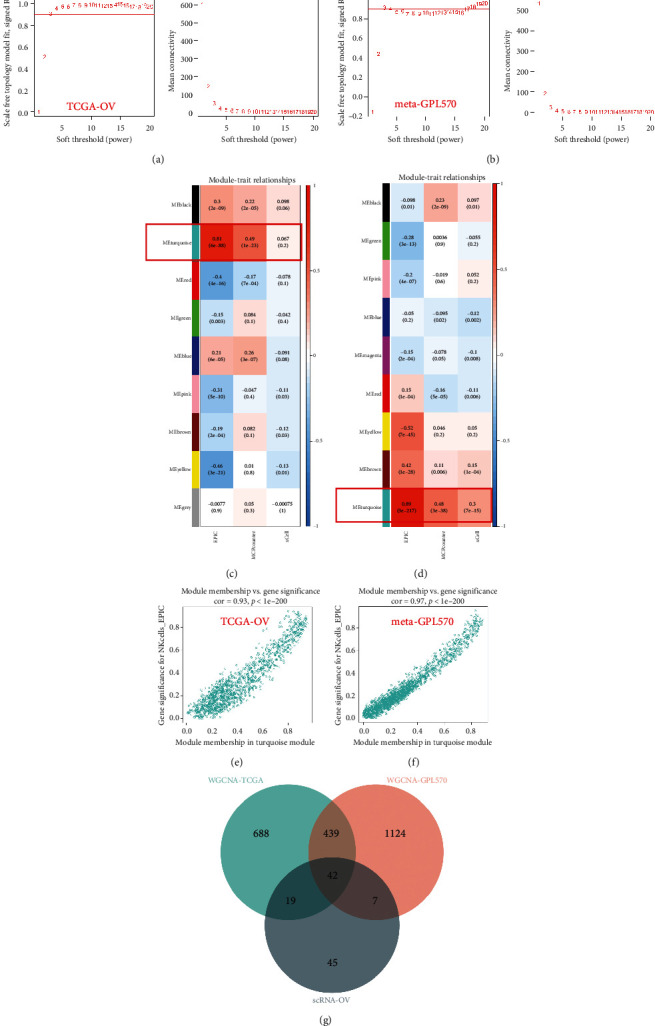
The WGNCA algorism identified NK cell marker genes (a, b) Scale-free exponent analysis and average connectivity analysis of soft threshold powers. (c, d) The heat map displayed the correlation between module characteristic genes and NK cell marker genes. (e, f) The correlation between module characteristic genes and NK cell EPIC. (g) The venn diagram showed overlapping genes for three screening datasets. A total of 42 genes were identified as NK cell-related marker genes.

The WGCNA network was built by first calculating the soft threshold power and then improving the coexpression similarity for the adjacency calculations. A topology analysis of the network is undertaken using the pickSoftThreshold function in the R package “WGCNA”. Based on the scale independence reaching 0.9 and the average connectivity being relatively high in both TCGA-OV and meta-GPL570 cohorts, the soft threshold power was set at 3 (Figures 3(a) and 3(b)).

**Figure 4:**
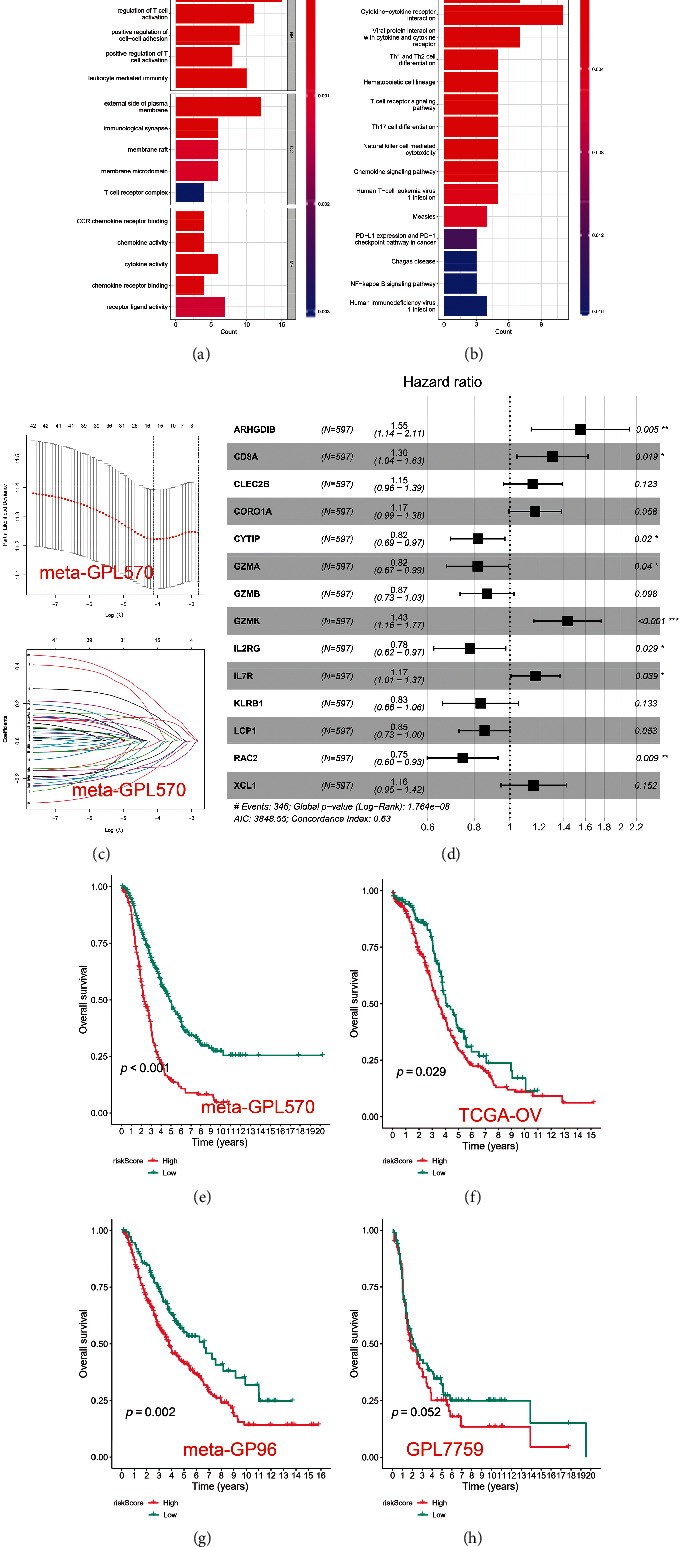
Construction of prognostic model based on the NK cell maker genes. (a, b) The GO and KEGG analyses of 42 NK cell marker genes. (c) The LASSO regression was used to reduce gene dimension, and 16 genes were screened for further analysis. (d) The multivariate COX regression analysis was used to obtain the coefficient of 14 genes in prognostic model. (e–h) K-M survival analysis of the prognostic model in different cohorts.

In the TCGA-OV and meta-GPL570 cohorts, we associate the modules with the immune infiltration algorithm and search for the most important associations. The results of this analysis showed that the module turquoise was significantly associated with NK cell infiltration (Figures 3(c) and 3(d)). In addition, the NK cell EPIC score we constructed was positively correlated with the turquoise module, which was 0.93, 0.97 in TCGA-OV, and meta-GPL570 cohorts, respectively (Figures 3(e) and 3(f)). Subsequently, we intersected the NK cell marker genes obtained based on the single-cell transcriptome data analysis with the NK cell marker genes obtained based on the WGCNA algorithm to obtain a total of 42 NK cell-related marker genes (Figure 3(g)). We performed pathway enrichment analysis on the 42 NK cell-related marker genes. GO analysis revealed NK cell-associated genes associated with T cell activation, leukocyte-mediated immunity, and immunological immunology (Figure 4(a)). KEGG analysis revealed these genes' enrichment in primary immunodeficiency, Th1 and Th2 cell differentiations, and natural killer cell-mediated cytotoxicity signal pathway (Figure 4(b)).

### 3.3. Establishment of Prognostic Model Based on 14 NK Cell Marker Genes

In order to predict the survival for each patient, we constructed a prognostic analysis based on 42 NK cell marker genes. We first used the meta-GPL570 cohort as a training set for LASSO regression analysis and screened 16 genes for further analysis (Figure 4(c)). Finally, we conducted the multivariate Cox regression analysis to optimize prognostic features, including only 14 of the most predictive genes (Figure 4(d)).

(2) $$\begin{array}{c} \text{Risk score}=0.44\times \text{ARHGDIB}+0.27\times \text{CD}8\text{A}+0.14\times \text{CLEC}2\text{B}+0.16\times \text{CORO}1\text{A}-0.20\times \text{CYTIP}-0.20\times \text{GZMA}-0.14\times \text{GZMB}+0.36\times \text{GZMK}-0.25\times \text{IL}2\text{RG}+0.16\times \text{IL}7\text{R}-0.18\times \text{KLRB}1-0.16\times \text{LCP}1-0.29\times \text{RAC}2+0.15\times \text{XCL}1. \end{array}$$

By ranking risk scores from high to low, patients were divided into low-risk and high-risk groups (low risk : score < median, high risk : score > median). Patients with a high-risk score had significantly shorter OS than patients with a low-risk score, according to Kaplan-Meier analysis (Figure 4(e)). Subsequently, TCGA-OV, meta-GP96, and GPL7759 external cohorts were used to verify the feasibility of the constructed predictive model (Figures 4(f)–4(h)).

### 3.4. Correlation between Risk Score and Tumor Microenvironment

**Figure 5:**
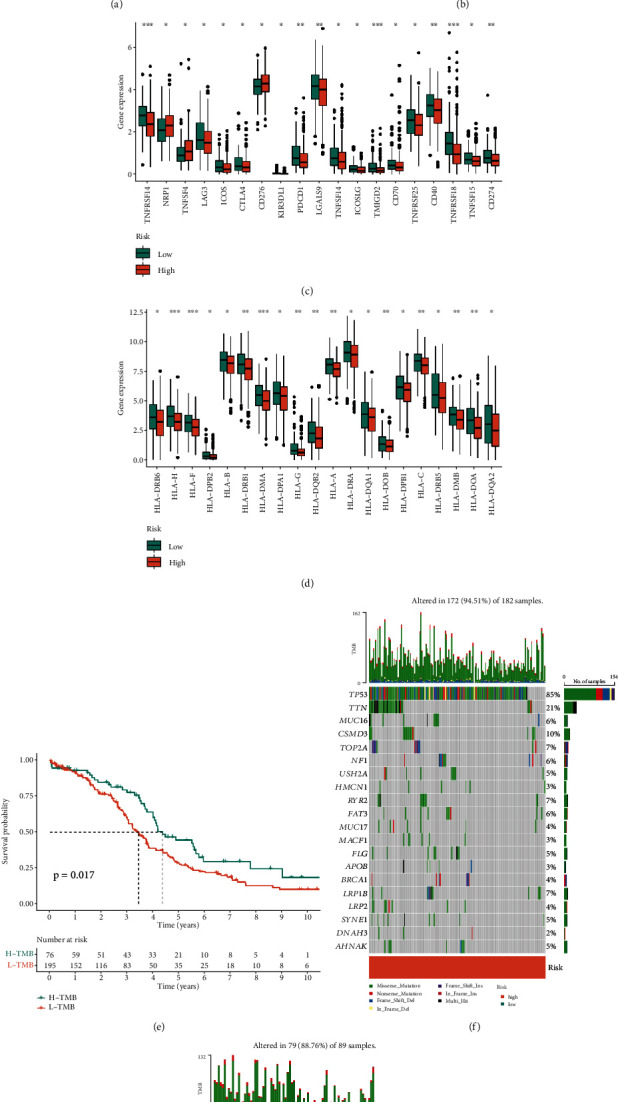
Tumor microenvironment assessment in different risk groups based on prognostic model. (a) Heat map showing/depicting immune cell infiltration landscape in high- and low-risk groups based on 5 algorithms. (b) Scatterplots showed the association of risk scores with StromalScore and ImmuneScore. (c, d) The boxplots showed the expression levels of immune regulators in the high- and low-risk groups. (e) K-M survival curves showed a survival difference between patients with high TMB and patients with low TMB. (f, g) The mutation landscape of high- and low-risk groups.

Since NK cells play an important role in antitumor immunity, we explored the relationship between the different risk based on the prognostic model and immune cell infiltration in OV patients. We employed TIMER, CIBERSORT, MCPcounter, xCell, and EPIC immune infiltration assessment algorithms to predict the proportion of immune cell infiltration in patients with high- and low-risk groups. M2 macrophages, cancer-associated fibroblast, hematopoietic stem cell, and stromal score were highly infiltrated in the high-risk group. NK cell, cytotoxicity score, B cell, and T cell CD4+Th1 were highly infiltrated in low-risk patients (Figure 5(a)). By using the ESTIMATE algorithm, we found that the risk score was positively correlated with StromalScore and negatively correlated with ImmuneScore (Figure 5(b)). Subsequently, we examined the expression of immunoregulators in patients with high- and low-risk groups. We found that immunoregulators and HLA families were generally highly expressed in the low-risk score group, while NRP1, TNFSF4, and CD276 were the opposite (Figures 5(c) and 5(d)). Therefore, we speculate that there were differences in immune cell infiltration and tumor mutation load between the two groups. For this reason, we divided patients into H-TMB and L-TMB according to tumor mutation burden (TMB). Medium TMB was 1.736842. Result turned out that patients with H-TMB had better survival outcomes than patients with L-TMB (Figure 5(e)). Figures 4(f) and 4(g) show that the frequency of mutation in patients with high-risk score (94.51%) was higher than that in patients with low-risk score (88.76%).

### 3.5. Predictive Model for Drug Sensitivity in OV Patients

**Figure 6:**
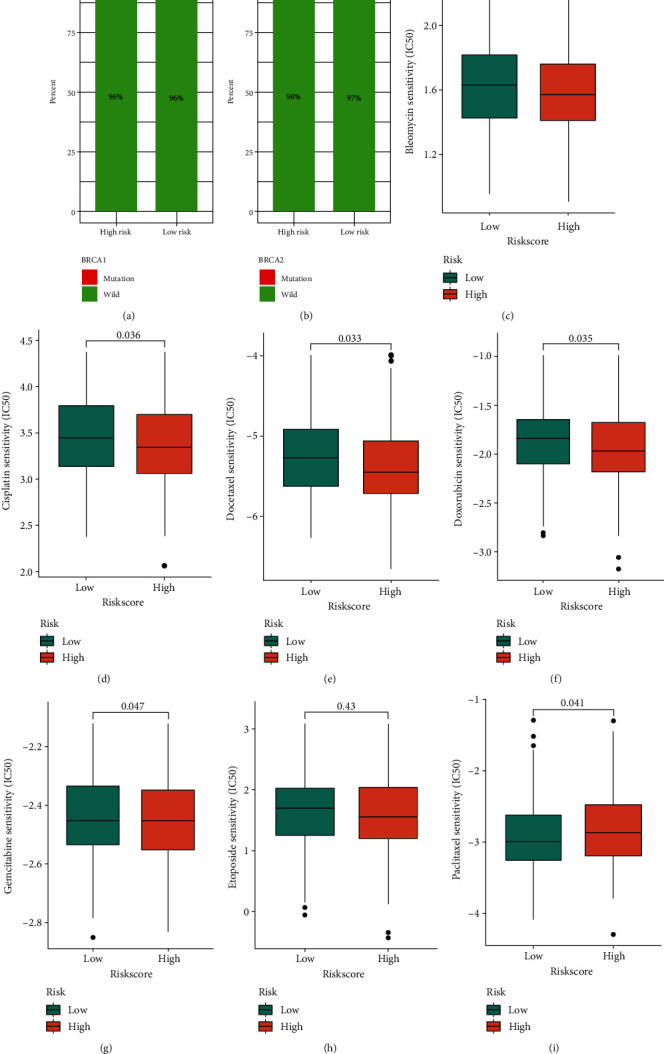
Drug sensitivity analysis. (a, b) The mutation patterns of BRCA1 and BRCA2 in high- and low-risk groups. (c–i) The IC_50_ of chemotherapeutic drugs in patients with the high- and low-risk groups.

We detected the mutation frequency of BRCA1 and BRCA2 between the high- and low-risk subgroups and found no difference between the two groups (Figures 6(a) and 6(b)). Subsequently, we performed drug prediction for patients in the high- and low-risk groups. We found that bleomycin, cisplatin, docetaxel, doxorubicin, gemcitabine, and etoposide were more effective in the high-risk group. Paclitaxel had a better therapeutic effect on patients in the low-risk group (Figures 6(c)–6(i)). Overall, these findings promoted a prognostic model as a biomarker for predicting individual drug sensitivity.

## 4. Discussion

As scRNA-seq technology developed rapidly, researchers began to focus more on the molecular characteristics of immune cells that infiltrate tumors. Despite that, most of the current research focuses on adaptive immune cells, ignoring the role of innate immune cells, which may have significant effects on clinical outcomes and immunotherapy response. Tumor-infiltrating NK cells were closely related to prognosis in patients with different solid tumors [25]. In the recent study by Shimasaki et al., NK cell marker genes were used to evaluate the infiltration of NK cells into TME, and the increased NK score significantly stratified the prognosis of patients with metastatic cutaneous melanoma [26]. Under the guidance of the above research, we employed three algorithms to observe the role of the NK cell score for predicting clinical outcomes of patients with ovarian cancer in two data sets and found that the prediction performance according to NK cell score was great. However, due to the algorithm being based on bulK RNA sequencing data, there was a certain deviation. Therefore, we obtained NK cell marker genes by combining scRNA-seq data and bulk RNA-seq data. Subsequently, we constructed a promising prognostic model based on NK cell marker genes for predicting clinical prognosis and immunotherapy efficacy and verified it in four independent cohorts. The high-risk score of the prognostic model was positively correlated with M2 macrophages, cancer-associated fibroblast, hematopoietic stem cell, and stromal score and negatively correlated with NK cell, cytotoxicity score, B cell, and T cell CD4+Th1. In addition, we found that bleomycin, cisplatin, docetaxel, doxorubicin, gemcitabine, and etoposide were more effective in the high-risk group, while paclitaxel had a better therapeutic effect on patients in the low-risk group.

In our investigation, the predictive prognostic model consisted of 14 NK cell marker genes (ARHGDIB, CD8A, CLEC2B, CORO1A, CYTIP, GZMA, GZMB, GZMK, IL2RG, IL7R, KLRB1, LCP1, RAC2, and XCL1), most of which were associated with prognosis or NK cell activity in OV patients. For example, Lado et al. identified two candidate genes belonging to the innate immune system: FCAR and CLEC2B. The CLEC2B gene was associated with NK cell and stimulated natural killer cells to play an immune defense mechanism [27]. Mace and Orange demonstrated for the first time that CORO1A promoted NK cells to exert cytotoxic functions and immune secretion by regulating F-actin breakdown, thus exerting the function of lytic immune effectors [28]. In addition, NK cells can kill gasdermin B- (GSDMB-) -enriched positive cells in tumor tissues by apoptosis mediated by granzyme A (GZMA), which is transcribed by the GZMA gene [29].To elucidate the molecular mechanisms of OV patients, laboratory experimental designs should focus on genes identified in the prognostic model.

This prognostic model has proven to be powerful predictive tools in training and validation cohorts. The excellent performance of the prognostic model has inspired us to investigate potential mechanisms. We first performed GO and KEGG analyses to explore the enrichment pathway of NK cell marker genes. GO analysis revealed NK cell-associated genes associated with T cell activation, leukocyte-mediated immunity. KEGG analysis revealed these gene enrichment in primary immunodeficiency, Th1 and Th2 cell differentiation, and natural killer cell-mediated cytotoxicity signal pathway. Poor prognosis in high-risk patients may be partly due to abnormal regulation of antitumor immunity, which was closely related to tumor proliferation and progression. Furthermore, tumor-infiltrating immune cells in TME play a crucial role in tumor development and have a significant impact on patient outcome [30]. We then compared ESTIMATE and CIBERSORT algorithms to determine the abundance of immune cell infiltration in high-risk and low-risk groups. The results showed that the level of immune cell infiltration in high-risk tumors was low, especially T cells and NK cells, suggesting that high-risk samples were called “cold tumors” and their antitumor activity was reduced [31]. The infiltration of immune cells in low-risk tumors can promote tumor cells to evade immune surveillance and promote tumor progression, which may partially explain the significantly reduced survival rate of patients with a high-risk score.

Our study still has some limitations. First, the expression and prognostic role of genes selected for prognostic models at the protein level warrants further investigation. A second limitation of our study is that the candidate genes we observed are all NK cell markers, and there is a high degree of spatial heterogeneity in the tumor immune microenvironment. Thus, the prognostic ability of the signature is restrictive. Finally, all mechanistic analyses in our study are descriptive. Future studies must explore potential mechanisms between prognostic model-associated gene expression and clinical outcome in OV patients. However, in the current prognostic model, our model still has great advantages, and NK cell signature genes also have their immune value. In addition, we used a large number of validation sets, which indicated the reliability and stability of our model.

## 5. Conclusion

In summary, we identified 14 genetic signatures based on NK cell marker genes and validated their strong predictive power for clinical outcome and response to chemotherapy in patients with OV. It can be used as a prognostic biomarker for clinical decision-making on individualized prediction and help to select suitable patients who can benefit from clinical treatment.

## Data Availability

All datasets generated for this study are included in the article material, including TCGA database (https://portal.gdc.cancer.gov/) and the GEO dataset (https://www.ncbi.nlm.nih.gov/gds/).
